# Geographic Distance Affects Dispersal of the Patchy Distributed Greater Long-Tailed Hamster (*Tscherskia triton*)

**DOI:** 10.1371/journal.pone.0099540

**Published:** 2014-06-09

**Authors:** Huiliang Xue, Min Zhong, Jinhui Xu, Laixiang Xu

**Affiliations:** 1 College of Life Sciences, Qufu Normal University, Qufu, Shandong, China; 2 Department of Biological Sciences, Auburn University, Auburn, Alabama, United States of America; East Carolina University, United States of America

## Abstract

Dispersal is a fundamental process in ecology influencing the genetic structure and the viability of populations. Understanding how variable factors influence the dispersal of the population is becoming an important question in animal ecology. To date, geographic distance and geographic barriers are often considered as main factors impacting dispersal, but their effects are variable depending on different conditions. In general, geographic barriers affect more significantly than geographic distance on dispersal. In rapidly expanding populations, however, geographic barriers have less effect on dispersal than geographic distance. The effects of both geographic distance and geographic barriers in low-density populations with patchy distributions are poorly understood. By using a panel of 10 microsatellite loci we investigated the genetic structure of three patchy-distributed populations of the Greater long-tailed hamster (*Tscherskia triton*) from Raoyang, Guan and Shunyi counties of the North China Plain. The results showed that (i) high genetic diversity and differentiation exist in three geographic populations with patchy distributions; (ii) gene flow occurs among these three populations with physical barriers of Beijing city and Hutuo River, which potentially restricted the dispersal of the animal; (iii) the gene flow is negatively correlated with the geographic distance, while the genetic distance shows the positive correlation. Our results suggest that the effect of the physical barriers is conditional-dependent, including barrier capacity or individual potentially dispersal ability. Geographic distance also acts as an important factor affecting dispersal for the patchy distributed geographic populations. So, gene flow is effective, even at relatively long distances, in balancing the effect of geographic barrier in this study.

## Introduction

Population persistence strongly depends on its own evolutionary capacity which in turn relies on the genetic variability. The capacity of genetic variability within and among populations results from many processes involving mutation, dispersal, genetic drift and selection. Dispersal refers to the movement of an organism from one place to another, which plays a fundamental role in population biology and conservation because it influences the genetic structure as well as the persistence of populations [1]. Dispersal could restrict the genetic differentiation in yellow warblers [2], and it tends to be considered as one of the main causes in maintaining the high genetic diversity of rodent populations [3].

It is well known that dispersal is conditional-dependent. There are a number of potential driving forces identified for dispersal including kin competition, inbreeding, resource competition and environmental stochasticity [1], [4]. How these factors work for dispersal varies among species according to their life histories and how they interact with the environment. Dispersal needs costs, which are important for the success of dispersal. The costs on dispersal are paid during dispersal movements [5] or prior invested for increasing the dispersal capacity [6]. For most animals, the cost and benefit of dispersal vary in space and time as well as among different species. The profitability of dispersal as a life history strategy varies, and a plastic dispersal strategy is expected to accommodate to this variation [7], [8].

Some previous studies have focused on the effects of several main factors on dispersal, such as population density [9]–[13] and sex [14], [15]. Changes in population densities lead to the changes in the social and competitive environment over time and will eventually cause dispersal. This is called density-dependent dispersal. The dispersal rates change as the density of the population change. Specifically, empirical and demographical data provided the evidence that negative density-dependent dispersal is prevalent in voles [9]–[11], while positive density-dependent dispersal is proposed in rodents[12], [13]. In particular groups of animals, the propensity to disperse has sex bias with different dispersal rates between males and females. For examples, most mammals show male-biased dispersal pattern meaning that males disperse more frequently and farther than females [14], whereas the female-biased dispersal mainly occurs in birds [15].

Geographic distance is also an important factor affecting dispersal. As the costs of movement increase with distance, a successful dispersal is often considered to occur when the distance between patches decreases [16]. The isolated distance of a patch, which is apart from other patches, will strongly impact the cost of dispersal as costs of movement increase with travel distance. Whether dispersal propensity is actually sensitive to the isolated degree of a patch depends on the ability to estimate the isolated degree. Isolation could potentially be assessed by several different methods [17]. Exploratory movement is one of prevalent method to assess the location of suitable habitat, depending on the inter-patch distance and the movement capacity of the animal. The perception of suitable habitat is important to estimate the distance to other patch without actually travelling the full distance [17], which was supported by data collected from field studies [18], [19]. The butterfly *Maniola jurtina* uses a non-random, systematic dispersal strategy and can detect and orient towards habitat from distances of 100–150 m [17]. The genetic and geographic distance between populations are generally positively correlated [20]–[23], suggesting an isolation-by-distance effect. However, several studies have suggested no correlation existing between geographic and genetic distances [24]–[28]. In our study, we expected to investigate the relationship between the geographic and genetic distance for the patchy-distributed hamster populations to better understand how geographic distance affect the population dispersal.

**Figure 1:**
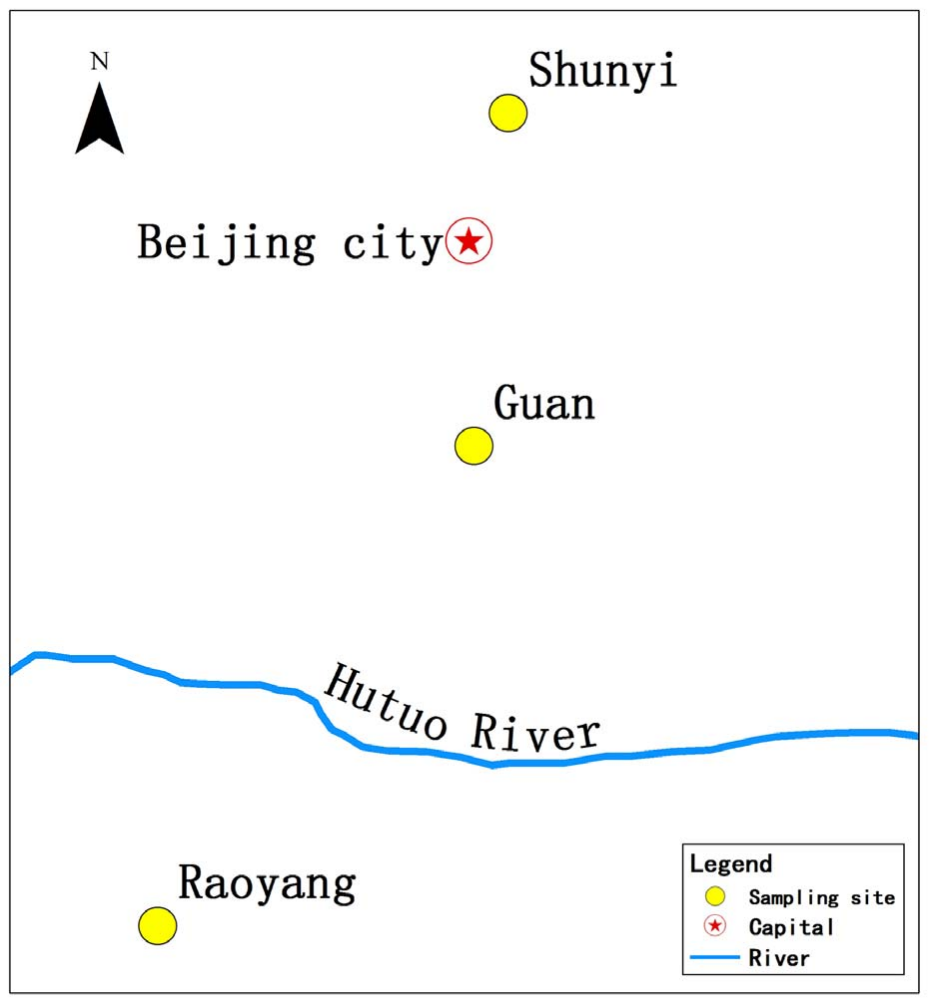
The sampling sites in the croplands of Raoyang, Guan and Shunyi Counties of the North China Plain and the potential barriers between the sampling sites.

In addition to distance, geographic barriers are also thought to be the important factors affecting dispersal [12]. Because of the dramatically increased use of land, habitat fragmentation is more and more obvious. Habitat fragmentation can increase the probability of local extinctions by destroying effective metapopulation structures. Rivers [29], roads [30]–[32] and valleys [33] all act as geographic barriers for the dispersal of some animal populations, but not for others. For example, rivers may act as physical barriers limiting the dispersal from one edge to the other for northern cavefish [34] and white-tailed deer [29], while they don't work for Chimpanzee [35] and Euglossini [36]. This divergence may be determined by the differences of dispersal abilities of particular animals or populations [36]. In this study, we chose three patchy-distributed Greater long-tailed hamster populations which were significantly isolated by Hutuo river and Beijing city respectively (Fig.1). Hutuo River is the main water source of Shijiazhuang County, Hebei Province, and it is 513.3 km in length and 46,000 square kilometers in watershed area. The river stores water annually and could be a potential physical barrier for dispersal. Beijing is the capital of the People's Republic of China and the center of politics, culture, education and international exchange. Beijing bears a great amount of people with lots of constructions and transportations which could restrict the dispersal of the Greater long-tailed hamster.

The Greater long-tailed hamster (*Tscherskia triton*) is widely distributed in croplands of North China [37], and it is one of dominant rodent species in the North China Plains [13]. Population abundance of the Greater long-tailed hamster varies greatly in space [13]. Here, we assessed the genetic differentiation and the gene flow among the three patchy-distributed Greater long-tailed hamster populations from Raoyang, Guan and Shunyi Counties of the North China Plain by using 10 polymorphic microsatellite loci, and to evaluate the role of geographic barriers and geographic distances for the dispersal to better understand the dispersal mechanism of animal populations. Rodents as an important functional group in ecosystem play an important role in the balance of ecosystem. Understanding the dispersal mechanism of the rodent population is very important for making reasonable and effective control methods.

## Materials and Methods

### Ethics statement

The Greater long-tailed hamsters in this study were captured in Raoyang, Guan and Shunyi Counties of the North China Plain, which were permitted by Wugong Station for Pest Monitoring and Forecasting, Hebei Province, China. All measures for the hamsters in this study were inspected and approved by Institutional Animal Care and Use Committee of the Institute of Zoology, Chinese Academy of Sciences (Permit Number: IOZ11012). All researchers had received appropriate training and affirmed before conducting animal studies.

### Sampling collection

The Greater long-tailed hamsters were captured using wooden-iron traps in Raoyang, Guan and Shunyi Counties of the North China Plain in autumn, 2000. The wooden-iron traps are box-shaped, with a wooden-side at the bottom and iron sheets with holes at other sides. One small side of the traps could be open or closed, and the other sides are fixed. The wooden-iron trap works by the leverage principle. The trap is 20 cm in length, 12 cm in width and 14 cm in height. The wooden side as bottom is baited with peanuts and equipped with spring device. When the Greater long-tailed hamsters step on the wood plate to eat and the spring set up release, the hamsters will be closed in the trap and then euthanized by CO_2_ asphyxiation immediately. These operations were approved by the Institute of Zoology, Chinese Academy of Sciences. Captured hamsters were numbered, sexed, weighed, dissected, and measured to provide estimates of body size, age, and reproductive condition [38]. Sampling sites Guan and Shunyi are isolated by Beijing city which is often considered as a physical barrier since it bears a great amount of people with lots of constructions and transportations. Guan and Raoyang sites are isolated by Hutuo River, which stores water annually and were potential barriers to dispersal for the Greater long-tailed hamsters (Fig. 1). More than 25 permanent plots were chosen in each sampling site, covering most crop types. Each plot contained two trapping lines and interval distance of 25–30 m. Twenty-five traps were placed along each line with an interval of 5 m. Trappings were conducted for 3 consecutive days two weeks followed by two weeks of non-trapping each month, to minimize the effects of removing too many animals. Based on the suggestion from a previous study that the samples should contain at least 20 individuals to obtain accurate estimates of genetic distance [39], we used ninety-three individuals in this study, including 30 from Raoyang, 31 from Guan and 32 from Shunyi. So, the sample size of each population in our study meets the requirements. To reduce the sampling errors, the distance between the sampling sites in the same population was more than 25 m.

### DNA extraction

Tissues were fixed in 90% ethanol, preserved in formalin solution and kept in the animal ecological Laboratory, Institute of Zoology, Chinese Academy of Sciences for more than 10 yrs. The genomic DNA was extracted from liver tissues using an improved phenol-chloroform extraction method [40]. DNA content is comparatively higher in liver tissue than others, and no fat matrix exist in liver tissue, therefore liver tissue is an ideal material for high DNA extraction efficiency. In order to prevent contamination in the process of DNA extraction, benches and plastic ware was cleaned with 10% bleach and sterile water and then exposed to ultraviolet (UV) light for 30 min. We used 10 extraction controls, none of which produced positive amplification during subsequent polymerase chain reaction (PCR).

### Genetic analyses

Ten microsatellite loci of the Greater long-tailed hamster (Table S1) were used on the basis of its high amplification efficiency and rich polymorphism [41]. The variation of each locus was examined by PCR containing 50 mM KCl, 10 mM Tris-HCl, 2.5 mM MgCl_2_, 0.2 mM each dNTP, 1 U of *Taq* DNA polymerase (Promega), 10 pM forward and reverse primers, and approximately 2 ng of template DNA in 25 µL. Amplifications began with a 5-min denaturing step at 94°C, followed by 30–35 cycles of the following thermal reaction: denaturing at 94°C for 45 s, annealing at 47∼55°C for 45 s, and extending at 72°C for 1 min, with a final extension for 5 min at 72°C. All products were analyzed in an ABI 377 instrument (Perkin-Elmer Applied Biosystems, Foster City, California) and the gel analysis was performed by using GENESCAN3.1 (Perkin-Elmer Applied Biosystem).

### Measures of Genetic Variation

Genetic variation within populations were assessed by using the measures including allelic richness (A_R_, number of alleles independent of sample size), Shannon's Information index (I), observed (H_O_) and expected (H_E_) heterozygosity. FSTAT version 2.9.2 [42] was used to calculate the measures for each locus. Hardy–Weinberg equilibrium tests were carried out by the Markov chain method [43], [44] in GENEPOP version 3.3[45]. Differentiation between various populations was estimated by the *F*_ST_ value [46].

An analysis of molecular variance (AMOVA) with Arlequin version 2.000 [47] was used to detect how much the genetic variance occupies the covariance components at various hierarchical levels. The three hierarchical levels were as follows: 1) within individuals, 2) among individuals within populations, and 3) among populations. The fixation indices *F*_IS_, *F*_IT_, and *F*_ST_ were calculated and the significant levels were tested respectively. Genetic distance [48] and gene flow [49] were calculated using the POPGENE version 1.31.

Geographic distances between sampling sites were calculated based on the approximate center of the sampling areas. Correlations between geographic distances and Nei's standard genetic distance [50] were calculated with the R-PACKAGE-module Mantel [51]. The statistical significance of the relationships was determined with 10 000 randomizations. The multiple regression analysis in the Mantel [51] were carried out to exclude the effects of the geographic barrier and the geographic distance on genetic differentiation for the three Greater long-tailed hamsters populations of Raoyang, Guan and Shunyi Counties.

## Results

### Genetic diversity

Among 10 microsatellite loci, there are 3 to 11 alleles with a mean of 6.1 alleles per locus (Table S1). Within populations, the mean number of alleles per locus ranged from 2.8 to 3.5 and allelic richness from 3.31 to 3.57 (Table 1). Observed heterozygosity was 0.557, 0.629 and 0.684 and expected heterozygosity was 0.601, 0.615 and 0.647 for Raoyang, Guan and Shunyi populations, respectively. No locus was found to deviate significantly from the Hardy-Weinberg equilibrium within each of three populations.

**Table 1:** Diversity indices calculated from microsatellites of three Greater long-tailed hamster (*Tscherskia triton*) populations in North China Plain.

Geographic populations	*N*	*A*	*A_R_*	*I*	*He*	*Ho*	*P_HW_*
Raoyang	30	3.5	3.566	1.9278	0.601	0.557	ns
Guan	31	2.8	3.314	1.8529	0.615	0.629	ns
Shunyi	32	3.3	3.474	1.9657	0.647	0.684	ns

*N*, Sample size;

*A*, average number of alleles/locus;

*A_R_*, allelic richness;

*I*, Shannon's Information index;

*He*, expected heterozygosity;

*Ho*, observed heterozygosity;

*P_HW_*, result of Hardy–Weinberg probability test for deviation from expected Hardy–Weinberg proportions.

Some alleles were more restricted, while others showed wide range of geographic distribution as shown in Table S2. Twenty-seven of 61 alleles were found only in one population, and not in the other two populations. For example, the alleles of 436, 438, 440, 444 and 450 at GYA66 locus were only detected in Raoyang population. The numbers of alleles observed in different geographic populations were 35, 28, 33 in Raoyang, Guan and Shunyi geographic population, respectively. There are 11, 4, 6, 5, 6, 6, 6, 9, 5 and 3 alleles at locus GYA66, GYA136, GYA183, GYA189, GYB13, GYB47, GYA185, GY103, GYB28 and GYA181 respectively for all the Greater long-tailed hamsters tested in this study.

Estimates of allelic richness (*A_R_*), Shannon's Information index (*I*), observed (*H*_O_) and expected (*H*_E_) heterozygosity for the microsatellites in each population are shown in Table S3. The mean values of the parameters *A_R_*, *I*, *Ho*, and *He* were 3.57, 1.93, 0.56 and 0.60 for Raoyang population, 3.31, 1.86, 0.63 and 0.62 for Guan population and 3.47, 1.97, 0.68 and 0.65 for Shunyi population, respectively. The parameters of *A_R_*, *I*, *Ho*, and *He* show that genetic diversity exists in Raoyang, Guan and Shunyi populations. No significant difference on the genetic diversity level was detected from the used parameters among the three populations.

### Genetic differentiation

Three pairwise estimates of *F_ST_* were significant. The highest *F_ST_* value (*F_ST_* = 0.0531, *P* = 0.0038) was observed between the populations of Raoyang and Shunyi. The lowest *F_ST_* value (*F_ST_* = 0.0327, *P* = 0.0041) was observed between the populations of Guan and Shunyi. The *F_ST_* value (*F_ST_* = 0.0359, *P* = 0.0027) between the populations of Raoyang and Guan was moderate. The *F_ST_* values of populations separated by the Hutuo River were higher than the ones separated by Beijing city,which indicates that the Hutuo River has higher barrier capacity than Beijing city for the Greater long-tailed hamsters. This leads to the higher genetic difference between the two sides populations of the Hutuo River than those of Beijing city.

The AMOVA showed that 71.6% of the variance is explained by within-individual variation, 8.9% by variation between individuals within population, and 19.5% by variation between populations. Although the total genetic variation is mainly from the variation of within-individuals, there was more than twice as much variation between populations as the one between individuals within populations. The overall *F*-statistics revealed the significance for *F_IS_* = 0.0281 (*P* = 0.0032), *F_IT_* = 0.0437 (*P* = 0.0053) and *F_ST_* = 0.0382 (*P* = 0.0039). Wright (1978) identified the problem of interpreting *F_ST_* values as an absolute value based on highly polymorphic loci and proposed that a *F_ST_*<0.05 could indicate a considerable population differentiation [52]. Our significant *F_ST_* value suggests that the genetic differentiation exists among three tested Greater long-tailed hamster populations.

### Correlations between geographic and genetic distance

**Figure 2:**
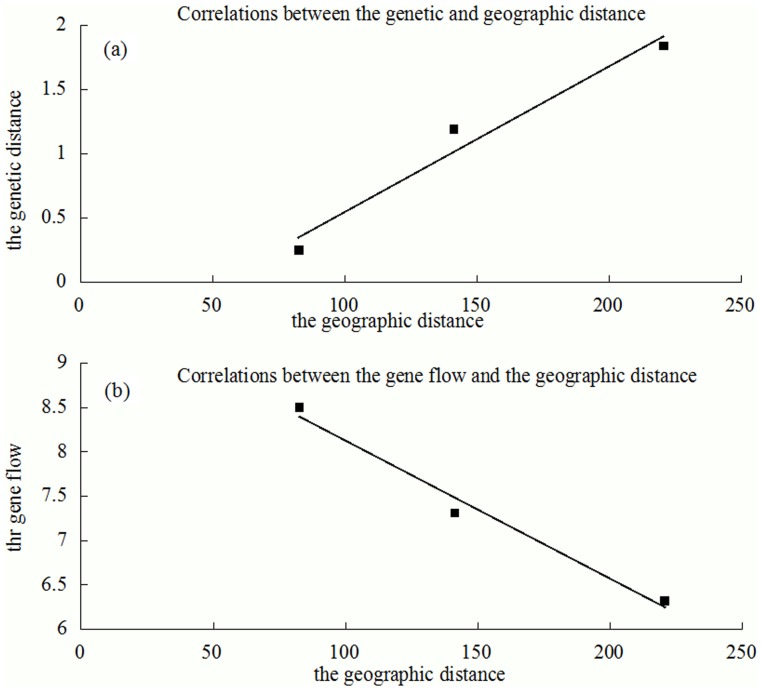
The correlations between the geographic distance and the genetic distance (a), and gene flow (b).

Genetic and geographic distances among the three examined patchy distributed geographic populations were summarized in Table 2. The genetic distance between the Raoyang and Shunyi populations (1.84) was found to be larger than the Guan and Shunyi (0.25) populations and the genetic distance between the Raoyang and Guan populations was moderate (1.19), suggesting a more distant genetic relationship between the Raoyang and Shunyi populations than the latter. This shows a direct proportional relationship between the genetic distance and the geographic distance (*r* = 0.98, *P* = 0.008), as shown in Fig.2.

**Table 2:** The geographic distance the genetic distance and the gene flow among the three examined geographic populations of the Greater long-tailed hamsters.

	Geographic distance (km)	Genetic distance	Gene flow
Between Raoyang and Guan	142.21	1.19	7.31
Between Raoyang and Shunyi	220.48	1.84	6.32
Between Guan and Shunyi	82.46	0.25	8.50

Gene flows between the different examined geographic populations were also summarized in Table 2. Higher level of gene flow exists between the Raoyang and Guan geographic populations than the Raoyang and Shunyi populations. The gene flow was inversely proportional to the geographic distance (*r* = −0.99, *P* = 0.017) as shown in Fig.2.

## Discussion

Generally, the populations that are isolated from one another by geographic barrier can evolve variable characters in order to adapt to local environments. Genetic variation of a population may lead to the character differentiation resulting from natural selection. To some extent analyzing the genetic structure of populations is very important to understand the population dynamics [53], [54]. The divergence of populations is usually investigated by using experimental genomic data [55], [56], as well as theoretical and empirical data [57], [58]. As a consequence, genetic variation was suggested to be the basis for populations to adapt to the environmental changes. Therefore, the importance of genetic diversity for the viability of populations is generally recognized [59]–[62].

From our study, we examined the genetic structure of three populations of Greater long-tailed hamster and found the significant genetic differentiation exist among these three patchy-distributed populations, suggesting the geographic distance plays an critical role in the genetic differentiation of these three patchy populations despite geographic barriers act on them to a certain degree. We have identified the correlation among genetic distance, gene flow and geographic distance and found that geographic distance is positively correlated with the genetic distance and is negatively correlated with the gene flow.

### Genetic diversity within populations

By using 10 microsatellites, we were able to detect comparable values of expected heterozygosity and allelic richness in three patchy distributed geographic populations. Mean allelic richnesses (*A_R_*) are 3.566, 3.314, and 3.474, and expected heterozygosities (*He*) are 0.601, 0.615, and 0.647, respectively for Raoyang, Guan and Shunyi populations. The values of the expected heterozygosity (*He*) for individual geese ranged from 0.38 to 0.51[63] and for noble scallop ranged from 0.119 to 0.459 [64]. The expected heterozygosities (*He*) detected in three populations are higher than those of the geese [63] and the noble scallop [64]. Therefore, we believe there is high genetic diversity existing within three patchy-distributed populations, which indicates high viability leading to fluctuation of hamster populations. At this point, our results are consistent with many previous studies [13], [14], [38], [65]. Gene flow between different populations is usually considered as an important factor leading to high genetic diversity within populations. The effective gene flow works for large populations with relative long geographic distances by balancing the effect of fragmentation [66]. In this study, our results also support the previous hypotheses that gene flow between different geographic populations could be critical resulting in high genetic diversity within populations, which is in accordance with our former study [13].

### Genetic differentiation and geographic distance effect

Natural selection, genetic drift and gene flow are main causes leading to genetic differentiation in populations. When populations are sufficiently isolated from one another selection and drift would enhance genetic differences, while oppositely, gene flow precludes their differentiation [67]. In large geographic areas, a variety of continuous differentiated populations would bring about from the interaction process of selection, genetic drift and gene flow, ranging from shared population to high degree differentiated populations [67]. Genetic differentiation may be a sign of the differences in ecological traits within a species' range, potentially leading to distinct ecotype-specific responses to different climates [68]–[70].

In this study, significant genetic differences were found among three geographic populations. High values of differentiation (*F_ST_* = 0.0531, 0.0327, and 0.0359) exist among the three geographic populations. Population differentiation can result from two genetic processes. 1) Populations may be isolated from one another by geographic barriers and therefore gene flow will be reduced among local populations. 2) Population differentiation level may increase with the increased geographic distance because the magnitude of gene flow declines with extended length of the geographic distance.

Geographic distance [16], [23] and geographic barriers [23], [71] are main factors affecting dispersal, which is an important cause affecting genetic variability [71], [72]. It is well known that geographic distance is negatively correlated with the dispersal in continuous distributed populations [20]–[23], but plays variable roles in patchy-distributed populations [24]–[28]. It is apparent that geographic barriers prevent dispersal. Studies have found that habitat discontinuities increase genetic differentiation in marine environments [73], [74]. Barriers, such as oceans, mountains, huge city communities, rivers etc., have more important effects on dispersal than geographic distance [71]. For example, rivers were identified as a major gene flow barrier for the army ant *Eciton burchellii* [75]. Sea lochs were the most important red deer gene flow barriers, followed by mountain slopes, roads and forests [71]. Former studies have shown that geographic distance and geographic barrier have prevented the dispersal at various levels among different species and barrier types. In this study, however, gene flow is effective, even at relatively long distances, in balancing the effect of geographic barrier under the interaction of geographic distance and geographic barriers.

Our study examined three geographic populations which are isolated by Beijing city or Hutuo river respectively as physical barriers, with far geographic distance among them. The correlation analyses among genetic distance, geographic distance and gene flow (Fig 2) showed significant isolation by distance despite the presence of gene flow. Thus, our results strongly agree with studies previously demonstrated [20], [21], [23] indicating the importance of analyzing the effects of distance on population differentiation. In this study, geographic distance explained 68.27% of the genetic differentiation, and only 31.73% of the genetic variance was explained by the geographic barrier, which suggests geographic distance has a significant effect on the dispersal of the patchy-distributed hamster populations, which is in accordance with former studies [20], [21], [23]. Therefore, geographic distance may partly account for the genetic differentiation among Raoyang, Guan and Shunyi populations, which was in accordance with the results obtained through former researches [76], [77].

However, several studies have suggested that no correlation exists between geographic and genetic distances among populations [24]–[28]. In this case, the geographic barrier has high capacity, even as absolute barriers and no gene flow exists between different geographic populations. While in our study, the barriers of Beijing city and Hutuo river are less restricted than what were expected.

Populations separated by the Hutuo river showed high genetic differentiation than populations on opposite sides of the Beijing city, suggesting that Hutuo river has higher barrier capacity than Beijing city for the Greater long-tailed hamster populations. In research of small mammal species, such as voles and ground beetles, barrier effect of roads to gene flow has clearly been shown [78], [79]. Human-made barriers, including highways and developed areas, act as absolute barriers for the populations of desert bighorn sheep (*Ovis canadensis nelsoni*) indicating no gene flow exists between the populations from two sides of the barriers [80]. Interestingly, gene flow of Alaskan brown bear (*Ursus arctos*) was found to be reduced or sometimes absent between four insular populations when separated by stretches of sea water of a few kilometres, but continuous gene flow between populations was detected when the stretches of sea water were much narrower at approximately 600 m [81], indicating that the span of sea water significantly affects the barrier capacity and the Alaskan brown bear can conquest the sea water barrier with width less than 600 m. Therefore, the restricted capacity of one physical barrier in various states could be different, even for the same species. Beijing is the metropolis of China possessing lots of roads which was considered to have high barrier capacity theoretically. Nevertheless, from our data, the less barrier capacity for Beijing city to hamster populations was indicated than that was expected. We speculate that some Greater long-tailed hamster may disperse by the underground path, so ground environment, including transportation and construction, has less barrier capacity, and the specific reasons need to be further studied.

## Conclusion

Genetic diversity and genetic differentiation exist in the three examined hamster populations. The barriers in this study acting on populations are less restricted than expected. Furthermore, the genetic differentiation is positively correlated with the geographic distance, while the gene flow shows a negative correlation. Geographic distance may act as one of main causes for the genetic differentiation of the patchy distributed hamster populations.

## Supporting Information

Table S1 Characterization of the microsatellite loci in Greater long-tailed hamster (*Tscherskia triton*).
(DOC)

Table S2 Alleles and their frequencies for the ten microsatellite markers in the three *Tscherskia triton* populations.
(DOC)

Table S3 Genetic diversity indices within three *Tscherskia triton* populations at ten microsatellite markers.
(DOC)

Table S4 Genotypes for the Greater long-tailed hamsters examined in this study at 10 microsatellite loci.
(DOC)
