# Supplementary material for: Geographic Distance Affects Dispersal of the Patchy Distributed Greater Long-Tailed Hamster (Tscherskia triton)
Source: PLoS One. 2014 Jun 9;9(6):e99540. doi: 10.1371/journal.pone.0099540 (PMC4049827; doi:10.1371/journal.pone.0099540)
Supplement: Table S1 — Characterization of the microsatellite loci in Greater long-tailed hamster (Tscherskia triton). (DOC) [file pone.0099540.s001.doc]

Table S1

| Locus | GenBank  Accession no. | Primer sequence(5´-3´) | *Ta*(°C) | *N_A_* | range of allele sizes (bp) | *He* | *Ho* | *P_HW_* |
| --- | --- | --- | --- | --- | --- | --- | --- | --- |
| GYA66 | AY780301 | F: CCCAGGAATGTTTATC | 53 | 11 | 436-474 | 0.68 | 0.51 | ns |
|  |  | R: AAGCCACCTTACTGACCC |  |  |  |  |  |  |
| GYA136 | AY780304 | F: CAGTCAGCCTTGTTCCAG | 54 | 4 | 148-184 | 0.27 | 0.35 | ns |
|  |  | R: CAAATGCCCTCTTAGTGT |  |  |  |  |  |  |
| GYA183 | AY780297 | F: GAACTGATGCCCTTGTGG | 51 | 6 | 340-368 | 0.38 | 0.41 | ns |
|  |  | R: CATTCCCTTATTGTCTGG |  |  |  |  |  |  |
| GYA189 | AY780309 | F: AAACATAAATGGGAGACA | 55 | 5 | 254-274 | 0.66 | 0.62 | ns |
|  |  | R: CTAAACCTGAACTGAGC |  |  |  |  |  |  |
| GYB13 | AY864068 | F: ATGAAGGTAGAAAGAGGGAA | 47 | 6 | 114-146 | 0.80 | 0.67 | ns |
|  |  | R: TTATGAGTGGGGTGCTGA |  |  |  |  |  |  |
| GYB47 | AY864074 | F: ATCCCTCTTCTCTCTTCTGG | 54 | 6 | 292-338 | 0.33 | 0.31 | nd |
|  |  | R: AAAGCACTACTACCTCTGA |  |  |  |  |  |  |
| GYA185 | AY780308 | F: AAACAGGAACTATGGAGGCA | 55 | 6 | 330-354 | 0.55 | 0.64 | ns |
|  |  | R: TGGTATAATTTATTTGGTG |  |  |  |  |  |  |
| GY103 | AY780298 | F: CTGGTCCTCTGAAAAG | 50 | 9 | 166-182 | 0.68 | 0.76 | ns |
|  |  | R: AACCTACTGCCTCTAT |  |  |  |  |  |  |
| GYB28 | AY864071 | F: CCTCTGTCATCCCCAAGT | 51 | 5 | 332-382 | 0.72 | 0.78 | ns |
|  |  | R: AGAAACCCTGTCTCAAAA |  |  |  |  |  |  |
| GYA181 | AY780305 | F: GGGCTGACTTACAGTTTTAG | 51 | 3 | 170-184 | 0.69 | 0.78 | ns |
|  |  | R: CAAGGTGGGCTTTGAGGT |  |  |  |  |  |  |

*Ta*, annealing temperature;

*N_A_*, number of observed alleles;

*He*, average expected heterozygosity;

*Ho*, average observed heterozygosity;

*P_HW_,* the results of probability tests for deviation from expected Hardy–Weinberg expectations.
