# Supplementary material for: Geographic Distance Affects Dispersal of the Patchy Distributed Greater Long-Tailed Hamster (Tscherskia triton)
Source: PLoS One. 2014 Jun 9;9(6):e99540. doi: 10.1371/journal.pone.0099540 (PMC4049827; doi:10.1371/journal.pone.0099540)
Supplement: Table S2 — Alleles and their frequencies for the ten microsatellite markers in the three Tscherskia triton populations. (DOC) [file pone.0099540.s002.doc]

**Table S2**

| Geographic populations | Locus | | | | | | | | | | Mean | s. d. |
| --- | --- | --- | --- | --- | --- | --- | --- | --- | --- | --- | --- | --- |
|  | GYA66 | GYA136 | GYA183 | GYA189 | GYB13 | GYB47 | GYA185 | GY103 | GYB28 | GYA181 |  |  |
| Raoyang | 436（15）  438（3）  440（13）  442（18）  444（8）  446（2）  450（1） | 148（43）  162（11）  184（6） | 340（8）  344（35）  348（17） | 266（6）  270（26）  274（28） | 114（8）  120（1）  128（40）  132（2）  140（1）  146（8） |  | 330（8）  336（12）  340（24）  348（16） | 166（8）  170（4）  172（18）  174（7）  176（11）  180（12） |  | 170（15）  178（38）  180（7） | 3.500 | 2.950 |
| Guan | 458（4）  462（45）  470（7）  474（6） | 162（3）  176（34）  184（25） | 348（17）  352（1）  360（20）  368（24） | 254（17）  260（34）  266（3）  270（8） |  | 292（14）  320（38）  338（10） | 332（24）  336（16）  348（22） | 168（28）  174（25）  180（9） | 332（18）  376（44） | 170（40）  178（22） | 2.800 | 1.420 |
| Shunyi | 444（6）  446（32）  470（7）  474（19） | 162（5）  176（15）  184（44） | 348（7）  352（7）  360（22）  368（28） | 254（16）  260（37）  266（11） |  | 300（11）  308（5）  318（17）  320（26）  338（5） | 332（18）  340（32）  354（14） | 170（31）  174（16）  178（6）  182（11） | 348（10）  356（14）  376（33）  382（7） | 170（48）  180（16） | 3.300 | 2.180 |
| Mean | 5.333 | 3.000 | 3.667 | 3.333 | 2.000 | 2.667 | 3.333 | 4.333 | 2.000 | 2.333 | 3.200 | 0.827 |
| s. d. | 1.556 | 0.000 | 0.222 | 0.222 | 8.000 | 4.222 | 0.222 | 1.556 | 2.667 | 0.222 | 6.100 | 4.890 |
| Tot. number | 11 | 4 | 6 | 5 | 6 | 6 | 6 | 9 | 5 | 3 |  |  |
