# Supplementary material for: Geographic Distance Affects Dispersal of the Patchy Distributed Greater Long-Tailed Hamster (Tscherskia triton)
Source: PLoS One. 2014 Jun 9;9(6):e99540. doi: 10.1371/journal.pone.0099540 (PMC4049827; doi:10.1371/journal.pone.0099540)
Supplement: Table S3 — Genetic diversity indices within three Tscherskia triton populations at ten microsatellite markers. (DOC) [file pone.0099540.s003.doc]

**Table S3**

| Locus | Raoyang | | | | Guan | | | | Shunyi | | | |
| --- | --- | --- | --- | --- | --- | --- | --- | --- | --- | --- | --- | --- |
|  | A_R_ | I | Ho | He | A_R_ | I | Ho | He | A_R_ | I | Ho | He |
| GYA66 | 5.5340 | 2.6623 | 1.0000 | 0.8927 | 2.5563 | 1.7198 | 0.5194 | 0.5633 | 3.8209 | 2.2361 | 1.0000 | 0.7558 |
| GYA136 | 2.7946 | 1.7800 | 0.5000 | 0.5503 | 3.1475 | 1.8422 | 0.6839 | 0.6431 | 2.8737 | 1.7968 | 0.7625 | 0.5737 |
| GYA183 | 3.2814 | 1.9404 | 0.2000 | 0.6712 | 4.0363 | 2.1537 | 1.0000 | 0.7816 | 3.9985 | 2.2128 | 0.6312 | 0.7771 |
| GYA189 | 3.4064 | 1.9483 | 0.5333 | 0.6944 | 3.5323 | 2.0950 | 0.6484 | 0.7150 | 3.3459 | 1.9660 | 0.7938 | 0.7828 |
| GYB13 | 3.0761 | 3.0575 | 0.8667 | 0.7271 | 1.0000 | 0.0000 | 0.0000 | 0.0000 | 1.0000 | 0.0000 | 0.0000 | 0.0000 |
| GYB47 | 1.0000 | 0.0000 | 0.0000 | 0.0000 | 4.7176 | 3.3460 | 0.7774 | 0.8430 | 4.6056 | 3.4191 | 0.9125 | 0.8341 |
| GYA185 | 5.4615 | 2.3095 | 0.9333 | 0.8232 | 4.9210 | 2.0846 | 0.6161 | 0.7684 | 4.6528 | 2.0358 | 0.9750 | 0.7329 |
| GY103 | 6.0139 | 2.6939 | 0.7000 | 0.9141 | 3.5799 | 2.0054 | 0.7774 | 0.7224 | 3.9811 | 2.2223 | 0.7875 | 0.7751 |
| GYB28 | 1.0000 | 0.0000 | 0.0000 | 0.0000 | 2.7009 | 1.6024 | 0.3935 | 0.5188 | 3.8563 | 2.2061 | 0.7562 | 0.7602 |
| GYA181 | 4.0955 | 2.8865 | 0.8333 | 0.7316 | 2.9493 | 1.6801 | 0.8742 | 0.5950 | 2.6000 | 1.5623 | 0.2250 | 0.4810 |
| Mean | 3.5663 | 1.9278 | 0.5567 | 0.6005 | 3.3141 | 1.8529 | 0.6290 | 0.6151 | 3.4735 | 1.9657 | 0.6844 | 0.6473 |
| s. d. | 1.3636 | 0.5807 | 0.3695 | 0.2891 | 0.8039 | 0.3811 | 0.2854 | 0.2169 | 0.7764 | 0.4206 | 0.3129 | 0.2195 |

A_R_, allelic richness (number of alleles independent of sample size); *I*, Shannon's Information index; *H*_O_, observed heterozygosity; *H*_E_, expected heterozygosity
