# Supplementary material for: Geographic Distance Affects Dispersal of the Patchy Distributed Greater Long-Tailed Hamster (Tscherskia triton)
Source: PLoS One. 2014 Jun 9;9(6):e99540. doi: 10.1371/journal.pone.0099540 (PMC4049827; doi:10.1371/journal.pone.0099540)
Supplement: Table S4 — Genotypes for the Greater long-tailed hamsters examined in this study at 10 microsatellite loci. (DOC) [file pone.0099540.s004.doc]

**Table S4**

| Counties | code | GYA66 | GYA136 | GYA186 | GYA189 | GYB13 | GYB47 | GYA185 | GY103 | GYB28 | GYA181 |
| --- | --- | --- | --- | --- | --- | --- | --- | --- | --- | --- | --- |
| Raoyang | 1 | 440/444 | 148/148 | 348/348 | 270/270 | 114/128 | 292/292 | 330/336 | 170/170 | 332/332 | 170/178 |
|  | 2 | 440/444 | 148/148 | 348/348 | 266/270 | 128/128 | 292/292 | 336/340 | 172/172 | 332/332 | 170/178 |
|  | 3 | 436/438 | 148/184 | 344/344 | 274/274 | 128/132 | 292/292 | 340/348 | 174/176 | 332/332 | 178/178 |
|  | 4 | 436/444 | 148/148 | 340/340 | 270/270 | 114/128 | 292/292 | 330/330 | 166/166 | 332/332 | 170/178 |
|  | 5 | 440/444 | 148/148 | 348/348 | 270/270 | 114/128 | 292/292 | 336/340 | 172/172 | 332/332 | 170/178 |
|  | 6 | 436/444 | 148/148 | 340/340 | 270/270 | 114/128 | 292/292 | 330/336 | 166/172 | 332/332 | 170/178 |
|  | 7 | 440/444 | 148/148 | 344/344 | 270/274 | 128/128 | 292/292 | 340/348 | 174/176 | 332/332 | 170/178 |
|  | 8 | 440/444 | 148/148 | 348/348 | 270/270 | 114/128 | 292/292 | 330/336 | 166/172 | 332/332 | 170/178 |
|  | 9 | 444/458 | 148/162 | 344/344 | 270/274 | 128/128 | 292/292 | 340/348 | 174/176 | 332/332 | 178/178 |
|  | 10 | 436/450 | 162/162 | 344/344 | 274/274 | 128/146 | 292/292 | 340/348 | 180/180 | 332/332 | 178/180 |
|  | 11 | 462/470 | 162/162 | 344/344 | 274/274 | 128/146 | 292/292 | 340/348 | 180/180 | 332/332 | 178/180 |
|  | 12 | 436/450 | 162/162 | 344/344 | 274/274 | 128/146 | 292/292 | 340/348 | 180/180 | 332/332 | 178/180 |
|  | 13 | 436/450 | 148/184 | 344/344 | 274/274 | 128/146 | 292/292 | 340/348 | 176/180 | 332/332 | 178/180 |
|  | 14 | 440/444 | 148/148 | 348/348 | 266/270 | 120/128 | 292/292 | 336/340 | 172/172 | 332/332 | 170/178 |
|  | 15 | 436/450 | 162/162 | 344/344 | 274/274 | 128/146 | 292/292 | 340/348 | 180/180 | 332/332 | 178/180 |
|  | 16 | 436/450 | 148/184 | 344/344 | 274/274 | 128/146 | 292/292 | 340/348 | 166/166 | 332/332 | 178/180 |
|  | 17 | 436/444 | 148/148 | 348/348 | 270/270 | 114/128 | 292/292 | 330/336 | 166/172 | 332/332 | 170/178 |
|  | 18 | 436/438 | 148/162 | 344/344 | 274/274 | 128/128 | 292/292 | 340/348 | 174/176 | 332/332 | 178/178 |
|  | 19 | 436/450 | 148/184 | 344/344 | 274/274 | 128/146 | 292/292 | 340/348 | 176/180 | 332/332 | 178/178 |
|  | 20 | 436/450 | 148/184 | 344/344 | 274/274 | 128/146 | 292/292 | 340/348 | 176/180 | 332/332 | 178/180 |
|  | 21 | 440/444 | 148/148 | 344/344 | 270/274 | 128/128 | 292/292 | 340/348 | 174/176 | 332/332 | 178/178 |
|  | 22 | 440/444 | 148/148 | 344/348 | 270/274 | 128/128 | 292/292 | 336/340 | 172/172 | 332/332 | 170/178 |
|  | 23 | 436/444 | 148/148 | 340/340 | 270/270 | 114/128 | 292/292 | 330/330 | 166/172 | 332/332 | 170/178 |
|  | 24 | 440/444 | 148/148 | 348/348 | 266/270 | 128/128 | 292/292 | 336/340 | 172/172 | 332/332 | 170/178 |
|  | 25 | 436/450 | 148/184 | 344/344 | 274/274 | 128/140 | 292/292 | 340/348 | 176/180 | 332/332 | 178/178 |
|  | 26 | 436/438 | 148/162 | 344/344 | 274/274 | 128/132 | 292/292 | 340/348 | 174/176 | 332/332 | 178/178 |
|  | 27 | 440/444 | 148/148 | 348/348 | 270/270 | 114/128 | 292/292 | 336/340 | 170/170 | 332/332 | 170/178 |
|  | 28 | 440/444 | 148/148 | 344/344 | 270/274 | 128/128 | 292/292 | 340/348 | 174/176 | 332/332 | 178/178 |
|  | 29 | 440/444 | 148/148 | 340/344 | 270/274 | 128/128 | 292/292 | 336/340 | 172/172 | 332/332 | 170/178 |
|  | 30 | 440/444 | 148/148 | 340/344 | 266/270 | 128/128 | 292/292 | 336/340 | 172/172 | 332/332 | 170/178 |
| Guan | 1 | 458/458 | 176/176 | 348/368 | 260/260 | 114/114 | 292/320 | 332/336 | 168/168 | 332/376 | 170/178 |
|  | 2 | 446/458 | 176/184 | 360/380 | 254/270 | 114/114 | 320/338 | 348/348 | 174/180 | 376/376 | 170/178 |
|  | 3 | 458/458 | 176/184 | 360/380 | 260/260 | 114/114 | 128/128 | 332/348 | 168/174 | 376/376 | 170/178 |
|  | 4 | 458/458 | 176/176 | 348/368 | 260/260 | 114/114 | 292/320 | 332/336 | 168/174 | 332/376 | 170/178 |
|  | 5 | 458/470 | 176/184 | 360/380 | 254/270 | 114/114 | 128/128 | 336/348 | 174/180 | 376/376 | 170/178 |
|  | 6 | 458/458 | 184/184 | 348/368 | 260/260 | 114/114 | 292/320 | 332/348 | 168/174 | 376/376 | 170/178 |
|  | 7 | 446/458 | 162/184 | 348/352 | 254/266 | 114/114 | 320/338 | 348/348 | 174/180 | 376/376 | 178/178 |
|  | 8 | 458/474 | 176/184 | 360/380 | 254/270 | 114/114 | 320/338 | 348/348 | 174/180 | 376/376 | 170/178 |
|  | 9 | 458/470 | 176/184 | 360/380 | 254/270 | 114/114 | 320/338 | 336/336 | 174/180 | 376/376 | 170/178 |
|  | 10 | 446/458 | 162/184 | 360/380 | 254/266 | 114/114 | 320/338 | 348/348 | 174/180 | 376/376 | 170/178 |
|  | 11 | 446/458 | 162/184 | 360/380 | 254/266 | 114/114 | 320/338 | 348/348 | 174/180 | 376/376 | 170/178 |
|  | 12 | 458/470 | 176/184 | 360/380 | 260/260 | 114/114 | 128/128 | 336/348 | 168/174 | 376/376 | 170/178 |
|  | 13 | 458/458 | 176/176 | 348/360 | 254/260 | 114/114 | 292/320 | 332/332 | 168/168 | 332/332 | 170/170 |
|  | 14 | 458/470 | 176/184 | 360/380 | 254/270 | 114/114 | 320/338 | 336/336 | 174/180 | 376/376 | 170/178 |
|  | 15 | 458/474 | 176/184 | 360/380 | 254/270 | 114/114 | 320/338 | 348/348 | 174/180 | 376/376 | 170/178 |
|  | 16 | 458/458 | 176/184 | 360/380 | 260/260 | 114/114 | 128/128 | 332/348 | 168/174 | 376/376 | 170/178 |
|  | 17 | 458/458 | 176/176 | 348/360 | 254/260 | 114/114 | 292/320 | 332/332 | 168/168 | 332/332 | 170/170 |
|  | 18 | 458/458 | 176/176 | 348/368 | 260/260 | 114/114 | 292/320 | 332/336 | 168/174 | 332/376 | 170/178 |
|  | 19 | 458/470 | 176/184 | 360/380 | 254/270 | 114/114 | 320/338 | 336/336 | 174/180 | 376/376 | 170/178 |
|  | 20 | 458/458 | 184/184 | 348/368 | 260/260 | 114/114 | 128/128 | 332/348 | 168/174 | 376/376 | 170/178 |
|  | 21 | 458/458 | 176/176 | 348/360 | 254/260 | 114/114 | 292/320 | 332/332 | 168/168 | 332/332 | 170/170 |
|  | 22 | 458/458 | 176/176 | 348/360 | 254/260 | 114/114 | 292/292 | 332/332 | 168/168 | 332/332 | 170/170 |
|  | 23 | 458/470 | 176/184 | 360/380 | 254/270 | 114/114 | 128/128 | 336/348 | 174/180 | 376/376 | 170/178 |
|  | 24 | 458/458 | 184/184 | 348/368 | 260/260 | 114/114 | 292/320 | 332/348 | 168/174 | 376/376 | 170/178 |
|  | 25 | 458/470 | 176/184 | 360/380 | 260/260 | 114/114 | 128/128 | 336/348 | 168/174 | 376/376 | 170/178 |
|  | 26 | 458/458 | 176/176 | 348/368 | 260/260 | 114/114 | 292/320 | 332/336 | 168/168 | 332/376 | 170/178 |
|  | 27 | 458/458 | 176/176 | 348/360 | 254/260 | 114/114 | 292/292 | 332/332 | 168/168 | 332/332 | 170/170 |
|  | 28 | 458/458 | 176/176 | 348/360 | 254/260 | 114/114 | 292/320 | 332/332 | 168/168 | 332/332 | 170/170 |
|  | 29 | 458/458 | 176/176 | 348/368 | 260/260 | 114/114 | 292/320 | 332/336 | 168/174 | 332/376 | 170/178 |
|  | 30 | 458/458 | 184/184 | 348/368 | 260/260 | 114/114 | 128/128 | 332/348 | 168/174 | 376/376 | 170/178 |
|  | 31 | 458/458 | 184/184 | 348/368 | 260/260 | 114/114 | 292/320 | 332/336 | 168/174 | 332/376 | 170/178 |
| Shunyi | 1 | 458/474 | 184/184 | 368/368 | 254/260 | 114/114 | 300/320 | 332/340 | 170/174 | 356/376 | 170/170 |
|  | 2 | 458/474 | 176/184 | 360/368 | 260/260 | 114/114 | 318/318 | 332/340 | 170/174 | 356/376 | 170/170 |
|  | 3 | 458/474 | 184/184 | 368/368 | 260/260 | 114/114 | 308/320 | 332/340 | 170/174 | 356/376 | 170/170 |
|  | 4 | 458/470 | 184/184 | 360/360 | 254/254 | 114/114 | 300/320 | 332/340 | 170/170 | 348/348 | 170/170 |
|  | 5 | 458/470 | 184/184 | 360/360 | 254/260 | 114/114 | 300/320 | 332/340 | 170/170 | 348/356 | 170/170 |
|  | 6 | 458/474 | 184/184 | 368/368 | 254/260 | 114/114 | 308/320 | 332/340 | 170/174 | 356/376 | 170/170 |
|  | 7 | 458/474 | 176/184 | 360/368 | 260/266 | 114/114 | 318/320 | 340/354 | 170/174 | 376/382 | 170/170 |
|  | 8 | 444/458 | 162/162 | 348/352 | 260/266 | 114/114 | 320/338 | 340/354 | 178/182 | 376/376 | 180/180 |
|  | 9 | 458/474 | 176/184 | 360/368 | 260/260 | 114/114 | 318/320 | 340/340 | 170/174 | 376/382 | 170/170 |
|  | 10 | 446/458 | 162/184 | 348/352 | 260/266 | 114/114 | 320/338 | 354/354 | 182/182 | 376/376 | 180/180 |
|  | 11 | 458/474 | 176/184 | 368/368 | 260/260 | 114/114 | 318/318 | 332/340 | 170/174 | 356/376 | 170/170 |
|  | 12 | 458/474 | 176/184 | 360/368 | 260/266 | 114/114 | 318/320 | 340/354 | 178/182 | 376/382 | 170/180 |
|  | 13 | 444/458 | 176/184 | 348/352 | 260/266 | 114/114 | 320/338 | 340/354 | 178/182 | 376/376 | 180/180 |
|  | 14 | 446/458 | 162/184 | 348/352 | 260/266 | 114/114 | 320/338 | 354/354 | 182/182 | 376/376 | 180/180 |
|  | 15 | 444/458 | 176/184 | 348/352 | 260/266 | 114/114 | 318/320 | 340/354 | 178/182 | 376/376 | 180/180 |
|  | 16 | 458/474 | 176/184 | 360/368 | 260/260 | 114/114 | 318/318 | 332/340 | 170/174 | 356/376 | 170/170 |
|  | 17 | 458/474 | 184/184 | 368/368 | 254/260 | 114/114 | 300/320 | 332/340 | 170/174 | 356/376 | 170/170 |
|  | 18 | 458/474 | 176/184 | 360/368 | 260/260 | 114/114 | 318/320 | 340/340 | 170/174 | 376/382 | 170/170 |
|  | 19 | 446/458 | 162/184 | 348/352 | 260/266 | 114/114 | 320/338 | 340/354 | 182/182 | 376/376 | 180/180 |
|  | 20 | 458/470 | 184/184 | 360/360 | 254/254 | 114/114 | 300/300 | 332/340 | 170/170 | 348/348 | 170/170 |
|  | 21 | 458/474 | 176/184 | 348/352 | 260/266 | 114/114 | 318/320 | 340/354 | 178/182 | 376/376 | 170/180 |
|  | 22 | 458/474 | 176/184 | 360/368 | 260/266 | 114/114 | 318/320 | 340/354 | 170/178 | 376/382 | 170/180 |
|  | 23 | 458/474 | 176/184 | 360/368 | 260/260 | 114/114 | 318/320 | 340/354 | 170/174 | 376/382 | 170/170 |
|  | 24 | 458/474 | 176/184 | 360/368 | 260/266 | 114/114 | 318/320 | 340/354 | 170/174 | 376/382 | 170/180 |
|  | 25 | 458/474 | 184/184 | 368/368 | 254/260 | 114/114 | 308/320 | 332/340 | 170/174 | 356/376 | 170/170 |
|  | 26 | 458/474 | 176/184 | 360/368 | 260/260 | 114/114 | 318/318 | 332/340 | 170/174 | 356/376 | 170/170 |
|  | 27 | 458/470 | 184/184 | 360/360 | 254/254 | 114/114 | 300/300 | 332/340 | 170/170 | 348/348 | 170/170 |
|  | 28 | 458/470 | 184/184 | 368/368 | 254/260 | 114/114 | 300/320 | 332/340 | 170/170 | 356/376 | 170/170 |
|  | 29 | 458/474 | 176/184 | 368/368 | 260/260 | 114/114 | 308/320 | 332/340 | 170/174 | 356/376 | 170/170 |
|  | 30 | 458/474 | 184/184 | 368/368 | 254/260 | 114/114 | 308/320 | 332/340 | 170/174 | 356/376 | 170/170 |
|  | 31 | 458/470 | 184/184 | 360/360 | 254/260 | 114/114 | 300/320 | 332/340 | 170/170 | 348/356 | 170/170 |
|  | 32 | 458/470 | 184/184 | 360/360 | 254/254 | 114/114 | 300/320 | 332/340 | 170/170 | 348/348 | 170/170 |
